# Hybrid Versus Autochthonous Turkey Populations: Homozygous Genomic Regions Occurrences Due to Artificial and Natural Selection

**DOI:** 10.3390/ani10081318

**Published:** 2020-07-30

**Authors:** Maria Giuseppina Strillacci, Stefano Paolo Marelli, Guillermo Martinez-Velazquez

**Affiliations:** 1 Department of Veterinary Medicine, University of Milan, Via Festa del Perdono, 7, 20122 Milano, Italy; stefano.marelli@unimi.it; 2 INIFAP-Campo Experimental Santiago Ixcuintla, Apdo. Postal 100, Santiago Ixcuintla 63300, Mexico; martinez.guillermo@inifap.gob.mx

**Keywords:** run of homozygosity, SNPs, turkey, inbreeding, ROH, genome variability, selected breeds, autochthonous populations

## Abstract

**Simple Summary:**

In this study we investigate the genomic differentiation of traditional Mexican turkey breeds and commercial hybrid strains. The analysis aimed to identify the effects of different types of selection on the birds’ genome structure. Mexican turkeys are characterized by an adaptive selection to their specific original environment; on the other hand, commercial hybrid strains are directionally selected to maximize productive traits and to reduce production costs. The Mexican turkeys were grouped in two geographic subpopulations, while high genomic homogeneity was found in hybrid birds. Traditional breeds and commercial strains are clearly differentiated from a genetic point of view. Inbreeding coefficients were here calculated with different approaches. A clear effect of selection for productive traits was recorded.

**Abstract:**

The Mexican turkey population is considered to be the descendant of the original domesticated wild turkey and it is distinct from hybrid strains obtained by the intense artificial selection activity that has occurred during the last 40 years. In this study 30 Mexican turkeys were genomically compared to 38 commercial hybrids using 327,342 SNP markers in order to elucidate the differences in genome variability resulting from different types of selection, i.e., only adaptive for Mexican turkey, and strongly directional for hybrids. Runs of homozygosity (ROH) were detected and the two inbreeding coefficients (*F* and F_ROH_) based on genomic information were calculated. Principal component and admixture analyses revealed two different clusters for Mexican turkeys (MEX_cl_1 and MEX_cl_2) showing genetic differentiation from hybrids (HYB) (F_ST_ equal 0.168 and 0.167, respectively). A total of 3602 ROH were found in the genome of the all turkeys populations. ROH resulted mainly short in length and the ROH_island identified in HYB (*n* = 9), MEX_cl_1 (*n* = 1), and MEX_cl_2 (*n* = 2) include annotated genes related to production traits: abdominal fat (percentage and weight) and egg characteristics (egg shell color and yolk weight). *F* and F_ROH_ resulted correlated to each other only for Mexican populations. Mexican turkey genomic variability allows us to separate the birds into two subgroups according to the geographical origin of samples, while the genomic homogeneity of hybrid birds reflected the strong directional selection occurring in this population.

## 1. Introduction

Turkey (*Meleagris gallopavo*) was the only vertebrate animal to be domesticated in ancient North America (i.e., the combined North and Central American sub-continents) [1]. The species has undergone two main migratory process: in the 16th century turkeys have been introduced into Europe and from that time they underwent a quick diffusion in European counties [2]; in the 17th century European colonists brought them back to North America, where they were hybridized with the local wild eastern subspecies (*Meleagris gallopavo silvestris*) [3].

Turkeys have undergone a massive expansion since then, becoming the second source of poultry meat worldwide, in particular in developing countries. In the last 40 years turkey stocks have almost tripled, average meat production per bird doubled, and selection pressure for economically important traits such as egg production, meat quality and body weight were exponentially improved, showing the effects of intensive selection applied in turkeys [4]. A fast-growing meat commercial hybrid (HYB) has resulted from the strong directional selection. Mexican turkeys, always bred without directional selection, are characterized by large variability in plumage (type and color) and body weight. For native Mexican turkey populations, no structured selection program was developed and applied at a population level. Furthermore, in the traditional farming system, birds are not under direct reproductive control by humans, farmed in a complete free-range system and as such allowed to migrate, facilitating the genetic exchange across flocks, villages and regions [5]. 

Genomic research in turkeys quickly developed in the last years thanks to the availability of a reference genome [6] and individual sequences, which opened opportunities also in whole-genome SNP discovery. Studies characterizing the genetic/genomic variability of turkey populations using SNPs markers (including mtDNA) are still limited [7,8,9,10,11]. Among these studies, Marras et al. [8] analyzed for the first time runs of homozygosity (ROH) in commercial turkey hybrids but no study is available, at best of our knowledge, on any autochthonous turkey population not under such strong artificial selection. ROH are uninterrupted long genomic regions including homozygous genotypes in adjacent SNP markers inherited from a recent common ancestor. ROH are primarily caused by inbreeding and so they reflect the mating selection plan: recent inbreeding—long ROH; more ancient inbreeding—short ROH [12]. In addition to inbreeding level, ROH can reveal information about population history [13], effects of inbreeding on complex traits [14] and diseases [15].

The autochthonous turkeys’ populations genome has not still been explored for ROH characteristics. The aim of this study was to characterize genomic diversity and to estimate genomic inbreeding in Mexican turkey populations and then in commercial hybrids to provide information on genomic features. One of the targets of the research was to investigate the effects of selection in different breeding environments on the genome of these different turkey populations.

## 2. Materials and Methods 

### 2.1. Sampling and Population Genetic Diversity 

The genotypes of all 38 hybrids and 30 Mexican turkeys used in this study are the ones previously analyzed by [7] and were obtained with the Axiom^®^ Turkey Genotyping Array (Affymetrix, Santa Clara, CA, USA). As we analyze previously produced data, no ethical issue applies to data production in this research.

In order to use the same number of SNPs for each animal, and then to reduce the bias ascribable to missing genotyping in inbreeding coefficients calculation and in other statistics, the original SNPs (400,451) dataset has been filtered maintaining for the analysis only SNP with call rate of at least 0.99. The final SNP dataset included 327,342 markers (for all animals), mapped on the Turkey_5.0 (GCA_000146605.1) genome assembly.

On this data set the following analyses have been performed:

- Principal components analysis (PCA) based on allele genotypes using the SVS software (SVS) version 8.8.3 (Golden Helix Inc., Bozeman, MT, USA). The graphical visualization of PCA was obtained by the ggplot2 R package (https://CRAN.R-project.org/package=ggplot2) [16].
- Estimation of pairwise Fixation Index (i.e., Wright’s F-statistic F_ST_) using the dedicated module of SVS.
- Determination of the most probable number of ancestral populations with the ADMIXTURE v.1.3.0 software [17]. ADMIXTURE was run from K = 2 to K = 5, and the optimal number of clusters (K-value) was determined as the one having the lowest cross-validation error (CV). Data input files were generated for ADMIXTURE using PLINK software version 1.07 [18]. The R script suggested by ADMIXTURE procedure, was used to perform a graphical representation of ADMIXTURE results.

### 2.2. Identification of Runs of Homozygosity (ROH)

ROH analyses were performed using the consecutive method implemented in the DetectRuns package [19] of R software setting a minimum of 1000 kb in size in order to exclude short and common ROH derived from strong LD [20] and 150 homozygous SNPs. In addition, no heterozygote and no missing SNPs were allowed in the ROH, and a maximum gap between SNPs of 1000 Kb was predefined in order to assure that the SNP density did not affect the ROH. ROHs were then grouped into 3 classes of length: 0–2 Mb, 2–4 Mb, 4–8 Mb for descriptive statistics. 

ROH statistics were calculated across individuals in each turkey population. The genomic regions with the highest frequency in ROH (ROH_island), have been identified by selecting the most frequent SNPs (where SNPs in a ROH were found in at least 70% of the birds of each cluster). 

The full turkey genes set (Annotation Release 103) was downloaded from NCBI online Database [21] and genes were catalogued within the ROH_island using the “intersectBed” command of BEDTools software [22]. Only genes with an official gene name were considered. A gene ontology (GO) functional annotation and KEGG pathway analyses was performed using the Database for Annotation, Visualization and Integrated Discovery (DAVID) v6.8 [23]. Chicken Quantitative Trait Loci (QTL) Database (Chicken QTLdb) was utilized (using “Search by associated gene” option) to catalogue QTL overlapping the identified ROH [24]. 

### 2.3. Inbreeding Coefficients

Two genomic inbreeding coefficients for the all birds were estimated using the routine implemented within SVS (*F*)—based on the difference between the observed and expected numbers of homozygous genotypes—and within DetectRUNs (F_ROH_)—based on the ratio between the total length of all ROH identified in a sample and the genome length covered by SNPs (in our study on chromosomes from 1 to 30, corresponding to 901,342,009 bp).

## 3. Results

### 3.1. Population Genetic Diversity

**Figure 1:**
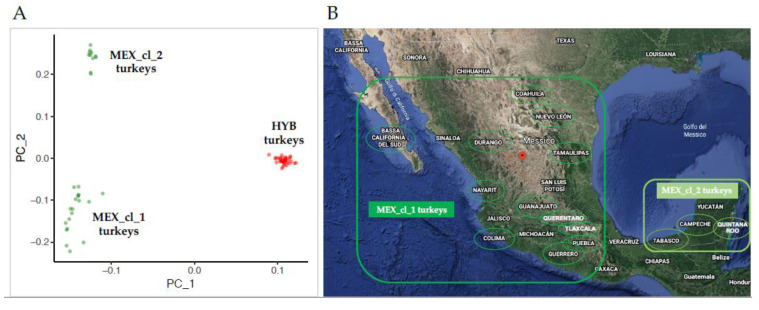
(**A**) PCA result: red dots = Hybrid turkeys; green dots = Mexican turkeys. (**B**) Mexico States involved in Mexican turkey sampling; map image has been modified from [25].

The PCA result is displayed in Figure 1A. On the first principal component (x axis–PC_1 eigenvalue = 5.33) a clear separation of HYB turkey samples (red dots) from the MEX ones (green dots) is observable. On the second principal component (y axis − PC_2 eigenvalue = 1.48) MEX turkeys is clustered in two well separated groups of 19 and 11 animals (MEX_cl_1 and MEX_cl_2, respectively in Figure 1A). As shown in Figure 1B the two clusters of Mexican samples reflect the geographical area of sampling.

These results are consistent with those obtained with the pairwise breed comparison using F_ST_. Although the F_ST_ values appear low, a genetic differentiation is found between HYB vs. MEX_cl_1 (F_ST_ = 0.168) and HYB vs. MEX_cl_2 (F_ST_ = 0.167). Differently, comparing MEX_cl_1 and MEX_cl_2 the F_ST_ value of 0.092 shows closer relatedness when comparing between these values and the one between the MEX and HYB populations.

**Figure 2:**
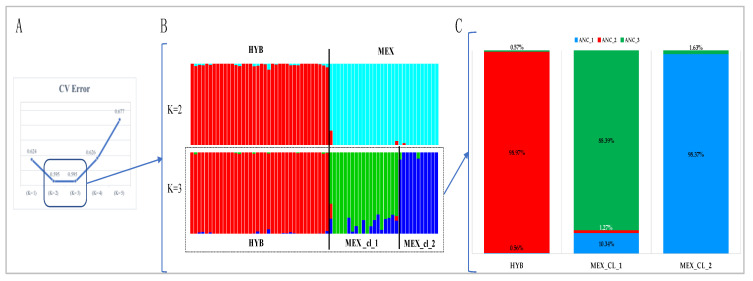
ADMIXTURE results. (**A**) Cross-validation error values (CV) plotted at K from 1 to 5: lowest CV values were identified at K = 2 and K = 3 (blue box). (**B**) K = 2 allowed ancestral distinction of hybrids (vertical red bars) from Mexican birds (vertical light blue bars); K = 3 was taken as the most probable number of inferred ancestors. Individual vertical bars are grouped by population (red–HYB, green–Mex_cl_1, and blue–Mex_cl_2). (**C**) Proportion of identified ancestral populations (ANC) calculated as the average of genetic ADMIXTURE score within each population at K = 3.

To investigate the ancestry composition of MEX and HYB turkeys, ADMIXTURE analysis was run on the entire data set for values of possible ancestors (K) between 2 and 5. The lowest CV value has been obtained with K = 2 and K = 3 values (both values = 0.595—Figure 2A). At K = 2, MEX populations (all animals) displays only one common ancestor for MEX_cl_1 and MEX_cl_2 (light blue), different from the HYB one (red bars). At K = 3 birds belonging to MEX_cl_1 (green bars) differ in ancestral composition from the ones of MEX_cl_2 (blue bars) and from those of HYB (red bars) (Figure 2B). Figure 2C shows the proportion of contribution of each ancestor to each of the three analyzed populations. All the populations appear to be mostly unique populations being represented by an ancestral genetic group in a proportion larger than 98% (HYB–ANC_2 in red and MEX_cl_2–ANC_1 in blue) and 88% for MEX_cl_1 (ANC_3 in green).

Considering PCA and ADMIXTURE results, ROH descriptive statistics were performed for MEX turkeys taking into account the samples’ clustering into two groups (MEX_cl_1 and MEX_cl_2).

### 3.2. Runs of Homozygosity (ROH)

A total of 3602 runs of homozygosity (ROH) were found in the entire turkey populations: 1809, 1438 and 355 runs in HYB, MEX_cl_1 and MEX_cl_2, respectively (Table 1, Table S1). The descriptive statistics of ROH across samples show clear differences among the three groups (Table 1). MEX_cl_1 shows both the largest number of ROH (170) and largest average length of ROH (1.911 Mb) per sample.

**Figure 3:**
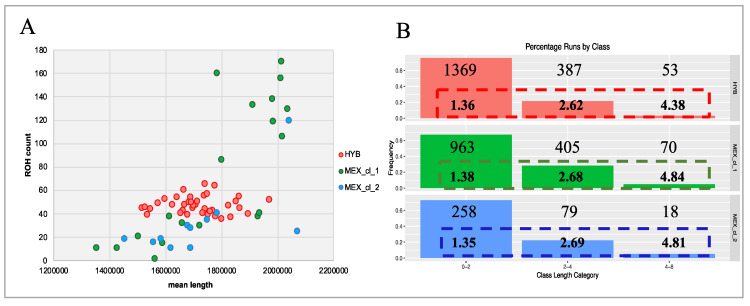
Graphical representation of runs of homozygosity (ROH) statistics. (**A**) Relationship between number and averaged total length (bp) of ROH in each bird; (**B**) Frequencies and counts of ROH for each class of length; the values in the dashed boxes represent the mean length of the ROH identified in each class of length.

Graphical representations of the ROH statistics for HYB and the two MEX subpopulations are shown separately in Figure 3. In details, Figure 3A shows the relationship between ROH count and the average total length of ROH for each individual. In general, the graphical distribution allows to identify three groups of birds: the first includes all turkeys of MEX_cl_2 (except 1) and part of MEX_cl_1. These samples showed a relatively low number of runs and a ROH mean length ranging from 1.4 to 2 Mb. Few birds overlap with the group of HYB that appear the most homogeneous showing limited variation for ROH count per individual (36 to 66) and a smaller variation in size of ROH respect to MEX birds. The third group includes one turkey of MEX_cl_2 and the remaining birds of MEX_cl_1, all characterized by a high count of ROH.

As shown in Figure 3B, ROH of < 2 Mb are the most frequent class in all turkeys being 1,369 (76%) in HYB, 963 (67%) in MEX_cl_1, and 258 (73%) in MEX_cl_2. Similar mean length values (dashed boxed in Figure 3B) were found in each group of birds within class of length.

Individuals exhibiting the lowest autozygosity belonged to the MEX turkey population (n. 3–MEX_cl_1 and n. 2–MEX_cl_1) showing a genome coverage of ROH encompassing less than 20 Mb. Instead, 8 samples of MEX_cl_1 and 1 sample of MEX_cl_2 with the highest autozygosity among all animals (i.e., inbred ones), had a total length of the genome covered by ROH larger than 200 Mb. HYB turkeys had on the other hand a more homogenous size of genome coverage by ROH among animals, ranging from 64 to 115 Mb. 

**Figure 4:**
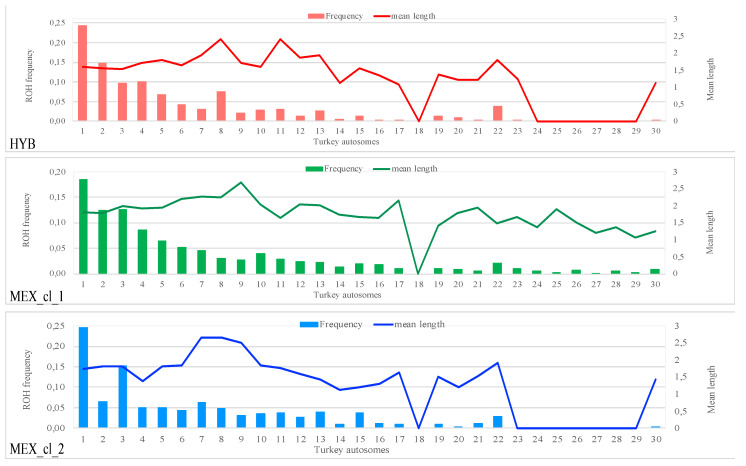
Frequencies (columns) and mean length in Mb (line) of ROH for each chromosome.

ROH were found only on some of the autosomes (Figure 4): 23 in HYB, 29 in MEX_cl_1, and 22 in MEX_cl_2. On chromosome 18 no ROH was identified in any of the analyzed populations. Chr 18 is short in length (332,615 bp) and includes the major histocompatibility complex (MHC locus B) [26] that it is well known to be a highly polymorphic locus involved in immune response. In HYB, no monomorphic SNPs resulted for the 62 markers mapping on this chromosome; instead, in MEX_cl_1 and MEX_cl_2, 24 and 19 SNPs were monomorphic even if nonconsecutive, thus not being a ROH. 

No correlation between chromosomes length and mean ROH length resulted in these populations (Figure 4).

**Figure 5:**
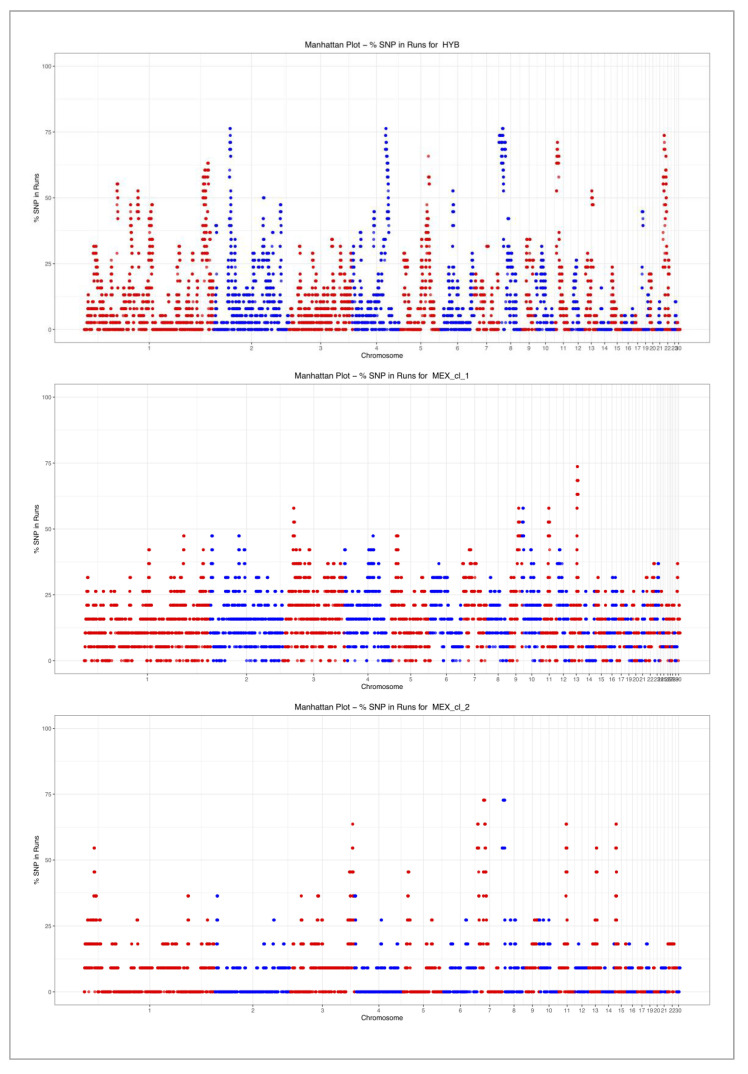
Proportion of SNPs in identified ROH for HYB, MEX_cl_1 and MEX_cl_2.

The graphs in Figure 5 show the proportion of SNP in ROH segments across the genome for the three groups of birds, HYB, MEX_cl_1 and MEX_cl_2.

A total of seven, one and two 2 ROH_islands (i.e., where SNPs in a ROH are found in at least 70% of the birds of each specific group) were identified in HYB, MEX_cl_1, and MEX_cl_2, respectively (Table 2). 

Table 2 also reports the list of genes (n. 69–HYB; n. 3–MEX_cl_1, and n. 46–MEX_cl_2) harbored in each identified ROH_island. An overlap was observed for HYB and MEX_cl_2 ROH_island on chr 8; all 17 genes mapping in the ROH_island of MEX_cl_2 are also included in the ROH_island of HYB.

Functional classification of genes annotated in turkey ROH_island provided by DAVID database is reported in Table S2.

The Animal Genome Database was accessed to identify if genes in ROH_island also overlapped in QTL annotated in database. Considering that there is no specific QTL database for the turkey species, we consulted the one available for chicken. 

Two genes are associated with four QTLs for production traits: the *BIRC5* gene on chr 7 (MEX_cl_2–eggshell color–QTL_ID:1914) and *RET* gene on chr 8 (HYB and MEX_cl_2–Yolk weight–QTL_ID: 62003 and 62004; Abdominal fat percentage–QTL_ID: 24505; Abdominal fat weight–QTL_ID: 24498).

Table 3 reports the list of genes (annotated in ROH_island) that have been associated with different traits in different species.

### 3.3. Inbreeding Coefficients (F-F_ROH_)

Genomic inbreeding parameters were calculated for all genotyped animals (Table 4). Proportions of homozygous SNPs were significantly different between the HYB and MEX populations: 58.5% vs. 76.6% (MEX_cl_1) and 72.7% (MEX_cl_2). 

**Figure 6:**
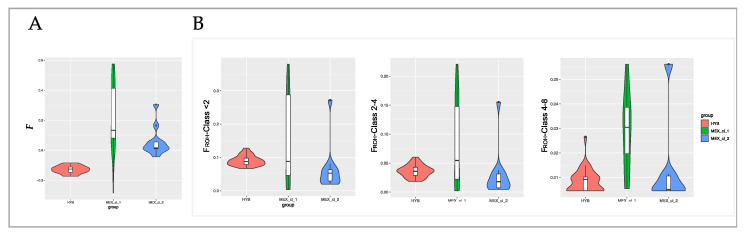
(**A**) Distribution of averaged *F* values. (**B**) Distribution of averaged F_ROH_ per class of length.

The inbreeding coefficients estimated using the proportion of homozygous SNP on all the autosomal SNP markers (*F*) were negative in HYB, positive for MEX_cl_1, and positive for MEX_cl_2 (averaged *F*-values are shown in Figure 6A). The minimum and maximum *F*-values were −0.17 and 0.08 for HYB, −0.284 and 0.57 for MEX_cl_1, −0.04 and 0.30 for MEX_cl_2.

F_ROH_ values were calculated based on the three classes of ROH length (Figure 6B) and on the total lengths covered by ROH. As displayed in Figure 6 the F_ROH_ values are different for each class of ROH length: F_ROH_ decrease with increasing of ROH classes of length in all three populations. In HYB the averaged F_ROH_ value clearly decreased with each length class, being the one of class 2–4 half of the value of Class < 2, and 5 times the value of Class 4–8. This drop was not so evident for Mexican turkeys F_ROH_ values. In addition, MEX_cl_1 individuals show higher average values and the highest number of outlier animals. The average genomic inbreeding based on the total observed ROHs (F_ROH_) is shown in Table 4. The minimum and maximum F_ROH_ values were 0.066 and 0.127 for HYB, 0.003 and 0.379 for MEX_cl_1, 0.020 and 0.272 for MEX_cl_2. Differences in F_ROH_ were found also along all chromosomes of all populations (Figure S1).

**Figure 7:**
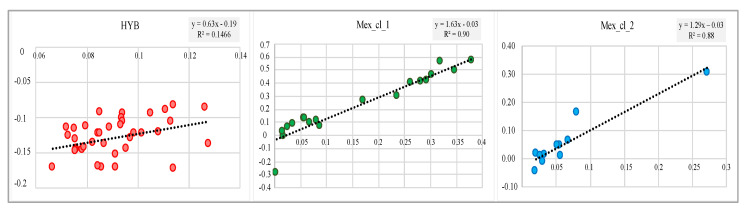
Regression and coefficient of determination (R^2^) calculated between *F* and F_ROH_. y = *F*; x = F_ROH_.

The linear relationship between *F* and F_ROH_ in HYB (Figure 7) is weaker (R^2^ = 0.15) compared to the one for MEX_cl_1 (R^2^ = 0.90) and Mex_cl_2 (R^2^ = 0.88).

## 4. Discussion

Turkeys, like most domestic animals, have undergone multi-generational changes influenced by controlled mating according to human’s needs and choices. In this study two different turkey populations (hybrid vs. autochthonous) were analyzed in order to compare the effects of more than 40 years of directional selection on heavy turkey hybrids’ genome structure vs a non-human controlled breeding of the Mexican turkeys farmed as a free-range population. According to the fact that no directional selection occurs in the MEX population, we can assume that only adaptive selection affected the genome structure of the two MEX groups of birds. These two Mexican turkey subpopulations allow a very interesting comparison between birds of the same origin, where the HYB, highly selected in the last 40 years, derives from ancestral birds originally brought into Europe by Spanish conquerors, and then brought back to America from Europe by settlers. Even if MEX and HYB belong to the same species, they represent two very well distinct groups: a wild type with no directional selection pressure (MEX) and a very heavily selected one (HYB). Their genomes then independently evolved for more than 500 years: the MEX adapted to challenging natural environmental conditions for more than five centuries; the HYB, in the last 40 years, heavily directionally selected by humans to best perform in artificial controlled environmental conditions.

Interestingly, the spatial distribution of Mexican turkeys resulted in two well clustered groups, as shown in PCA (Figure 1A). This bird’s distribution reflects the geographical sampling areas characterized by different environments: MEX_cl_1 birds were collected in seven Mexican States where mountain areas are present at several altitude (Baja California Sur, Coahuila, Nuevo Leon, Guerrero, Tlaxcala, Puebla, Tamaulipas, Guanajuato, Colima, Durango, Nayarit, Queretaro) and MEX_cl_2 birds were sampled in three Mexican States (Campeche, Tabasco, Quintana Roo), that are mainly flat and hilly areas as shown in the topographic physical map in Figure 1B. This result is also supported by the F_ST_ values and from the ADMIXTURE outcomes that clearly provided indication of different genetic background for the two subgroups of the Mexican turkeys. We speculate that the difference in genome may be the result of adaptation to the different environments of the two sampling areas of Mexico where the birds originated, lived and evolved. This may also be interpreted as the exchange of genetic material among states in the same geographical region, i.e., MEX_cl_1 and MEX_cl_2, but no exchange of birds across them. 

When analyzed according to ROH, the differences at genomic level among the three turkey groups are evident. Although the proportion of ROH within length classes is similar for Mexican turkeys and hybrids, the number of ROH, as well as the mean ROH length per sample, is different in MEX populations (Figure 3A,B) relatively to those identified for HYB that, in addition, appear less variable respect the MEX ROH. The two MEX clusters in fact exhibited both lowest and highest autozygosity (inbred animals) showing a ROH genome coverage encompassing less than 20 and ROH > 200 Mb, respectively. Instead, HYB turkeys had a genome coverage affected by ROH within a shorter range (64 to 115 Mb). Commercial HYB derives from the cross of heavily selected (and inbreed) lines, that we considered not related among them. As such their genome is expected to be highly heterozygous. Additionally, the genome of hybrid birds is the results of very few recombination events crossing the parental lines. A four-way cross (A × B × C × D) see only one generation of recombination event between A and B and between C and D. ROH are then identified when parental lines are homozygous for the same allele (SNP) in the same region of the genome. These regions, that in outbred populations represent sites of recent inbreeding, in HYB indicates regions under common selection in parental lines. According to this hypothesis, the ROH are then expected to be smaller and less variable in HYB and we can speculate that they harbor genes under common directional selection pressure in all parental lines. In MEX, where we assume no directional selection for productive performance, ROH are likely to indicate regions under adaptive positive selection and recent inbreeding possibly due to mating occurring among relatives in small flocks.

The *F* values listed in Table 4 and their distribution (Figure 6A) confirm that HYB birds exhibit a large proportion of heterozygous genotype respect the expectation under HW equilibrium (average *F* = −0.129). This is expected due to the assortative mating realized crossing parental inbred lines. Additionally, in HYB variation in *F* value is much lower than in MEX birds with MEX_cl_1 showing both larger inbreeding coefficients values (Table 4) and larger standard deviation (*F* = 0.227 +/− 0.23). The linear relationship between *F* and F_ROH_ shown in Figure 7 indicate that the homozygous SNPs are, for MEX, mainly in ROH, occurrence that is not realized in the same manner for HYB. These results could be explained by the higher number of heterozygous SNPs observed (Table 4) and by the lower proportion of homozygous SNPs distributed on genome outside of the ROH in HYB. In fact, this last proportion, calculated within samples as total number of homozygous SNPs defining ROH over the total observed homozygous SNPs, is lower in HYB respect to MEX birds: 0.88–HYB and 0.92 for MEX_cl_1 and MEX_cl_2.

Although individual variability exists in the ROH genomic location, regions with a high prevalence of ROH (ROH_island) were identified in all populations: seven in HYB, one in MEX_cl_1 and two in MEX_cl_2. Some of HYB regions (on chr 2, 4, 8, and 11) appear to overlap with the ones identified by [8] (according to the published Manhattan plot; no details on ROH_island provided by authors). In addition, the ROH_islandw identified on chr 8 partial overlap with those found in MEX_cl_2. No overlapping ROH_islandw have been identified between the two Mexican turkey groups, most likely because they ancestral origin is different, as shown by the ADMIXTURE results. 

Several genes harbored in ROH_island are known to be involved in expression for production, functional and health traits in different species (Table 3). Turkey gene annotation is still not as complete as in other species where the genes mapping in ROH here found are available. 

Except for *DYDC1* and *PCDH15* all other genes were identified in association with productive or adaptive traits in livestock species. 

Particularly, the genes mapping in ROH for HYB, except for *ARID5B* are related to several meat production efficiency traits. On the contrary genes for MEX are mainly related to adaptation to harsh environment characteristics. These findings clearly show that genome of the MEX birds, here considered the wild type, subjected to a different selection pressure respect to HYB. 

Among the four genes included in Table 3 for the chicken species, *PCDH15* and *TBC1D1* are those containing the largest number of homozygous SNPs. 

In particular, *TBC1D1*—which polymorphism has been related to several traits including glucose homeostasis, energy metabolism in skeletal muscle and blood, insulin resistance, adiposity, obesity, and type 2 diabetes [41,42]—seems to reflect the directional selection occurred in HYB turkeys. In support of this latter assumption, as reported for commercial broiler chickens, *TBC1D1* is located within a QTL explaining differences in growth between broilers and layers and a haplotype sweep regarding this gene is fixed in the high and low growth lines [43]. 

## 5. Conclusions

The evolution of the genome of Mexican turkey was shown to have occurred in a different manner respect to the one that has happened recently in the voluminous commercial turkey population. Even if the annotation in Turkey species is still at its infancy, genes in ROH_island can be ascribed to two groups of characteristics: production efficiency for HYB, and fitness to natural environment for MEX. The production efficiency in an artificial selection program, as the one occurring in HYB, can be ascribed to greater fitness. In fact, artificial selection is favoring the best performing animals in an artificially controlled environment for a desired trait, in the HYB meat production. This manuscript presents the first evidence that the comparison of a heavy selected *Meleagris gallopavo* strain versus an unselected one can disclose variations in genomic structure and identify regions in autozygosity due to different selection criteria.

## Figures and Tables

**Table 1:** Descriptive statistics for runs of homozygosity (ROHs) identified in HYB (Hybrids) and in the two MEX (Mexican) subpopulations (MEX_cl_1 and MEX_cl_2). Values are expressed in Mega base pair (Mb).

Pop	Samples	N. ROH	Min–Max (Mean) N. ROH per Sample	Min–Max (Mean) ROH Length per Sample
HYB	38	1809	36–66 (47)	1–6.970 (1.717)
MEX_cl_1	19	1438	2–170 (75)	1–7.652 (1.911)
MEX_cl_2	11	355	11–120 (32)	1–7.999 (1.825)

**Table 2:** Details of ROH_island defined by SNP occurrences (>70%). Start and End ROH positions as well as the ROH length are expressed in bp and are in concordance with the Turkey_5.0 (GCA_000146605.1) genome assembly.

Chr	Start ROH Position	End ROH Position	ROH Length	Gene ^1^
ROH_island in HYB				
2	23,019,543	23,992,770	973,227	MTA3, HAAO
4	48,508,445	49,752,492	124,4047	RBM47, CHRNA9, RHOH, N4BP2, PDS5A, UBE2K, SMIM14, UGDH, LIAS, RPL9, KLB, RFC1, WDR19, KLHL5, TMEM156, FAM114A1, KLF3, TBC1D1, PGM2, RELL1, C4H4orf19, NWD2
8	35,534	3,564,550	3,529,016	GHITM, NRG3, SH2D4B, TSPAN14, FAM213A, *EXOSC3, DYDC1, MAT1A, RASGEF1A, CSGALNACT2, RET, BMS1, PLAC9, ANXA11, ECD, FAM149B1, DNAJC9, TFAM, UBE2D1, CISD1, IPMK, PCDH15*
8	4,968,066	5,916,410	948,344	CHAT, OGDHL, PARG, NCOA4, GPRIN2, SYT15, FAM35A, GLUD1, ADIRF, SNCG, MMRN2, BMPR1A, LDB3, OPN4, WAPL
8	8,483,294	9,342,515	859,221	RTKN2, ARID5B, TMEM26, RHOBTB1, CDK1
11	3,058,621	3,097,354	38,733	-
22	1,595,252	1,986,192	390,940	ASIP, EIF2S2, RALY
ROH_island in MEX_cl_1				
13	11,244,459	11,371,128	126,669	HYDIN, MTSS1L, SF3B3
ROH_island in MEX_cl_2				
7	9,145,051	10,872,877	1,727,826	DNAH7, BIRC5, STK17B, HECW2, GTF3C3, C7H2orf66, PGAP1, ANKRD44, SF3B1, COQ10B, HSPD1, RFTN2, BOLL, PLCL1, SATB2, C7H2orf69, TYW5, MAIP1, SPATS2L, KCTD18, SGO2, AOX1, BZW1, CLK1, PPIL3, NIF3L1, ORC2, FAM126B
8	1,401,303	3,748,153	2,346,850	*EXOSC3, DYDC1, MAT1A, RASGEF1A, CSGALNACT2, RET, BMS1, PLAC9, ANXA11, ECD, FAM149B1, DNAJC9, TFAM, UBE2D1, CISD1, IPMK, PCDH15,* PRKG1

^1^ Italic: overlapped genes.

**Table 3:** Genes mapping in ROH_island in different species for different phenotypes.

Pop.	Gene	Phenotype	Species	References
MEX_cl_2	*ANKRD44*	Skin thickness	Swine	[27]
MEX_cl_2	*AOX1*	Residual feed intake	Bovine	[28]
HYB	*ARID5B*	Adaptive immunity	Human	[29]
HYB/MEX_cl_2	*DYDC1*	Acrosome biogenesis; Spermiogenesis	Mouse	[30]
MEX_cl_1	*HYDIN*	Thermal pain response	Mice	[31]
MEX_cl_1	*HYDIN*	Marbling score	Bovine	[32]
HYB	*MMRN2*	Meat juiciness	Bovine	[33]
HYB	*MMRN2*	Meat tenderness	Bovine	[34]
MEX_cl_2	*NIF3L1*	Skin thickness	Swine	[27]
MEX_cl_2	*ORC2*	Marbling score	Bovine	[32]
HYB/MEX_cl_2	*PCDH15*	Femoral head separation	Chicken	[35]
HYB	*PGM2*	Feed efficiency; Reduction of environmental footprint	Chicken	[36]
MEX_cl_2	*PLCL1*	Skin thickness	Swine	[27]
MEX_cl_2	*PRKG1*	Humoral response to *Mycobacterium avium* ssp. Paratuberculosis	Bovine	[37]
MEX_cl_2	*SF3B1*	Carcass merit and meat quality	Swine	[38]
HYB/MEX_cl_2	*TFAM*	CORT-induced fatty liver protection	Chicken	[39]
HYB	*TBC1D1*	Carcass	Chicken	[40]
HYB	*TBC1D1*	Growth and Serum Clinical-Chemical	Chicken	[41]
HYB	*TBC1D1*	Growth	Rabbit	[42]

**Table 4:** Descriptive statistics of homozygosity, heterozygosity and inbreeding coefficients (*F* − F_ROH_).

POP	Obs Hom	Exp Hom	Obs Het	Exp Het	*F* Mean (SD)	F_ROH_ Mean (SD)
HYB	58.5	63.3	41.5	36.2	−0.129 (0.025)	0.130 (0.015)
MEX_cl_1	76.6	69.8	23.4	29.4	0.227 (0.23)	0.161 (0.13)
MEX_cl_2	72.7	71.1	27.3	27.6	0.056 (0.10)	0.065 (0.07)

Obs Hom = Observed Homozygoty (%); Exp Hom = Expected Homozygoty (%); Obs Het = Observed Heterozygoty (%); Exp Het = Expected Heterozygoty (%).

## Supplementary Materials

The following are available online at https://www.mdpi.com/2076-2615/10/8/1318/s1, Table S1: List of all ROH identified in commercial hybrid and Mexican turkeys, Table S2: Functional classification of genes annotated in turkey ROH_island provided by DAVID database, Figure S1: F_ROH_ calculated on all chromosomes of all turkey populations.
