# Supplementary figures and images for: Hybrid Versus Autochthonous Turkey Populations: Homozygous Genomic Regions Occurrences Due to Artificial and Natural Selection
Source: Animals (Basel). 2020 Jul 30;10(8):1318. doi: 10.3390/ani10081318 (PMC7460020; doi:10.3390/ani10081318)

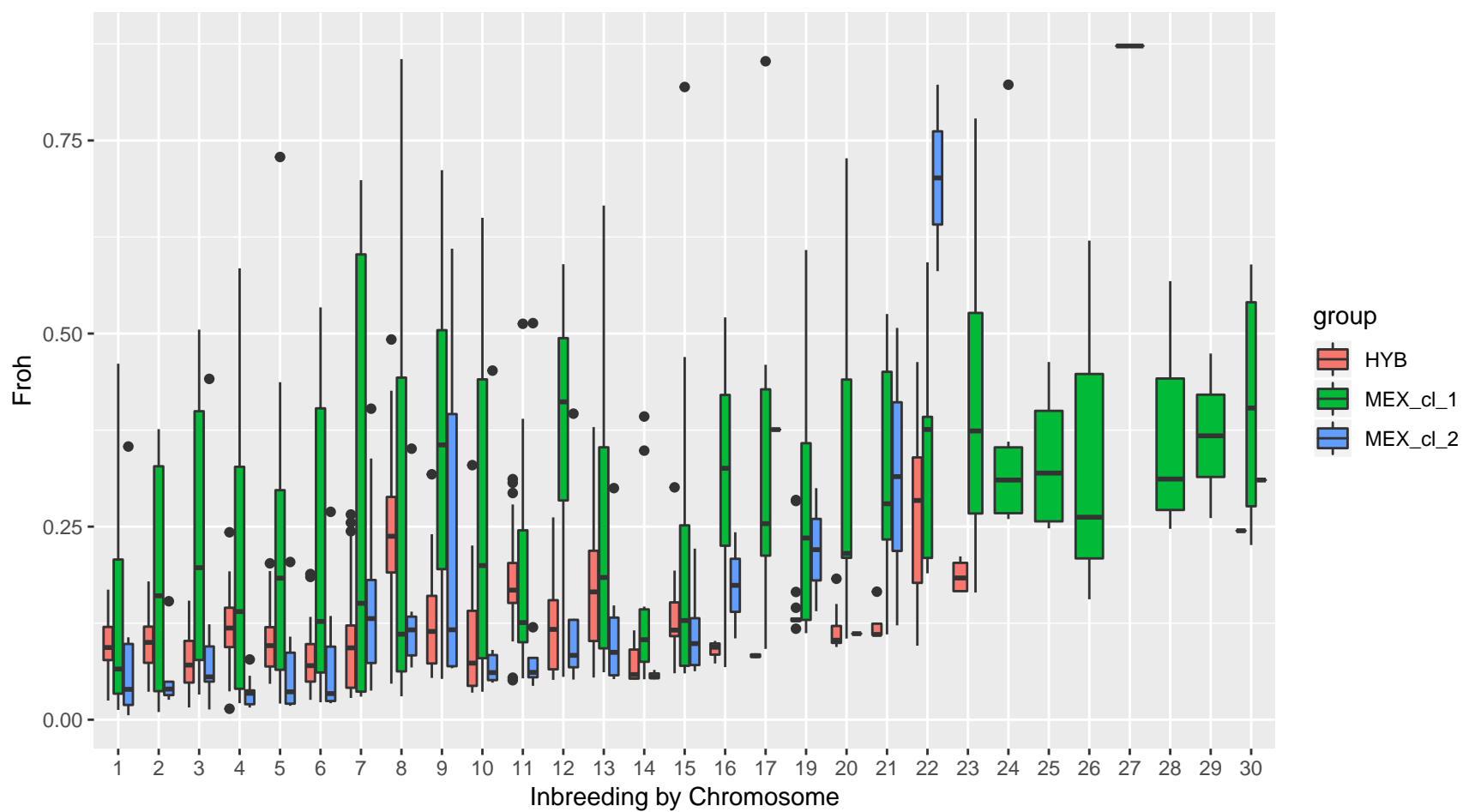

Supplement: Supplementary file 1 [file animals-10-01318-s001.zip › Supplementary_File/Figure_S1.pdf]
